# Stereotactic Radiosurgery for Trigeminal Neuralgia: A Retrospective Multi-Institutional Examination of Treatment Outcomes

**DOI:** 10.7759/cureus.554

**Published:** 2016-04-03

**Authors:** Raj Singh, Joanne Davis, Sanjeev Sharma

**Affiliations:** 1 Department of Radiation Oncology, Joan C. Edwards School of Medicine, Marshall University; 2 Clinical Programs, The Radiosurgery Society; 3 Department of Radiation Oncology, St. Mary's Medical Center

**Keywords:** srs, stereotactic radiosurgery, trigeminal neuralgia, registry, facial pain

## Abstract

Objectives

The purposes of this study are to assess the effectiveness of CyberKnife® stereotactic radiosurgery (SRS) in providing both initial and sustained pain relief for patients with both forms of trigeminal neuralgia (TN), assess potential prognostic factors, and examine treatment-related toxicities.

Methods

The RSSearch^®^ Patient Registry was screened for TN cases from July 2007 to June 2015. We evaluated initial pain relief achieved by examining changes in the Visual Analog Scale (VAS) scores following SRS. Prognostic factors relating to initial pain relief and the relationship between maximum dose (Dmax) and toxicity incidence were analyzed via univariate logistic regressions. We evaluated prognostic factors relating to sustained pain relief using the Kaplan-Meier method and log-rank analysis.

Results

Our analysis included 125 TN1 patients and 38 TN2 patients with initial VAS scores ≥ 3 treated at 16 community radiotherapy centers. Median Dmax for both cohorts was 75 Gy with a larger range for TN1 cases (67.42 Gy - 110.29 Gy) as compared to TN2 cases (70.00 Gy - 78.48 Gy). At initial follow-up, mean VAS scores after SRS were significantly lower for TN1 and TN2 patients (p < 0.0001). The vast majority of TN1 (87.2%) and TN2 (86.8%) patients experienced initial pain relief. Higher initial VAS scores (p = 0.015) were correlated with a greater likelihood of initial treatment success for TN1 patients. We did not identify any treatment or patient characteristics that had significant effects on initial pain relief for TN2 patients. Of the TN1 cohort, 28 of 125 patients reported follow-ups one year or greater after SRS. Twenty-three of 28 TN1 patients (82%) reported VAS scores of 1 or less at one-year follow-up, and eight of 11 patients (72%) had VAS scores of 1 or less at the two-year follow-up. No potential prognostic factors for long-term pain relief were significant. Roughly 18% and 11% of TN1 and TN2 patients, respectively, experienced acute toxicities (all RTOG Grade 1 or 2), with the most common being sensory neuropathy, generalized pain, and nausea. Dmax > 75 Gy was not a predictor of toxicity incidence in TN1 cases (p = 0.597) but was significant for TN2 patients (p = 0.0009 following Fisher's exact test).

Conclusions

SRS is an effective treatment option for TN patients in community settings. Initial pain relief following SRS was achieved in a vast majority of TN patients with associated minor toxicities observed in less than 20% of all patients.

## Introduction

Trigeminal neuralgia (TN) is a chronic, debilitating pain condition of the trigeminal nerve, which provides widely distributed sensory innervation to the face. TN has an incidence of 12 per 100,000 people, disproportionately affecting the elderly and female patients [1]. The classic form of the disease is TN1, which is associated with sudden shocks of facial pain that can last anywhere from a few seconds to minutes. The second form of the disorder is known as TN2 and, unlike TN1, is characterized by constant pain, generally of lower intensity.

The first step in the management of TN is pharmacotherapy, such as carbamazepine, gabapentin, and oxcarbazepine. Side effects relating to these regimens include impaired memory, nausea, insomnia, confusion, and peripheral neuropathy [1-3]. If such medications are found to be ineffective at pain management or lead to adverse side effects, then surgical techniques, notably rhizotomies or microvascular decompression (MVD), may be utilized. MVD is the most invasive of these two options as it requires both open craniotomy and hospitalization of the patient. Previous studies have demonstrated that MVD can provide long-lasting pain reduction with a success rate of 83.5% [4-5]. Complications of MVD include facial numbness, facial palsy, CSF leaks, hearing deficits, and incisional infections [6]. Alternatively, percutaneous or radiofrequency rhizotomies have been found to be effective at providing initial pain relief but are generally not associated with long-term control of TN [7-8]. Side effects have been noted in roughly 67% of patients, with the majority being minor sensory issues but others including dysesthesia, labial herpes, and severe sensory deficits. Blomstedt, et al. also found technical obstacles in about 47% of procedures with 8% of rhizotomies having to be paused due to major complications [9].

Stereotactic radiosurgery (SRS) has been a viable treatment option for TN since first being discussed by Leksell [10]. SRS is an outpatient, non-invasive alternative to rhizotomies or MVD following the failure of medical pain management or surgery with a lower risk of concurrent side effects [11-12]. The foundation of SRS for the treatment of TN comes from published studies using the Gamma Knife® (GK), a device used specifically for intracranial radiosurgery [13-14]. Over the last decade, the use of linear accelerators (LINACs) and robotic radiosurgery devices to treat TN has increased. However, there is a lack of data from multicenter studies reporting on the clinical utilization, optimal dose, pain control, and side-effects using LINAC-based SRS [15]. Similarly, there have been limited large retrospective multi-institutional studies examining treatment outcomes for TN using the CyberKnife^®^ stereotactic radiosurgery system (CK SRS).

The RSSearch^®^ Patient Registry, a multi-institutional international SRS/SBRT (stereotactic body radiation therapy) database with information on screening, treatments, and outcomes for over 15,000 patients, serves as an ideal resource to study the effectiveness of CK SRS for TN1 and TN2 management in community settings [16]. Our goals in this analysis are to evaluate the pain reduction achieved in both TN1 and TN2 populations following SRS, examine whether potential prognostic factors can be identified for a better likelihood of treatment success, and discuss the toxicities stemming from treatment.

## Materials and methods

Previous studies have described the methodology and design of the RSSearch^® ^Patient Registry  (Clinicaltrials.gov Identifier: NCT01885299) ^ ^[16]. Participation of centers in RSSearch^®^ is voluntary, and no compensation is provided to patients or participating centers. All principal investigators are provided RSSearch^®^ protocols, case report forms, sample patient informed consents, and web-based training for data entry and database navigation. Local Institutional Review Board/Ethics Committee (IRB/EC) approval is required at all participating centers, and informed consent was obtained from all patients, as required by individual IRB/ECs, prior to data entry.

As RSSearch^®^ is a registry, there were no pre-defined treatment criteria. All patients were treated using the CyberKnife^®^ Stereotactic Radiosurgery System. CyberKnife^®^ is a frameless, image-guided SRS system consisting of a LINAC mounted on a 6-axis robotic arm that delivers a high dose of radiation to a lesion of interest while minimizing damage to adjacent healthy tissue. *In situ *x-ray images allow for head-tracking during procedures with non-invasive head immobilization to provide homogenous, non-isocentric, and conformal delivery of radiation doses to intracranial targets, such as the trigeminal nerve [17].

All patients were treated with single fractions of radiation with specific treatment planning based on varying institutional imaging, contouring, and treatment guidelines. Given the different definitions of prescription doses from multiple institutions in our study, all prescriptions were converted to maximum dose points (Dmax) to allow for uniform comparison across centers.

Treatment outcomes were assessed using Visual Analog Scale (VAS) scores for facial pain that were reported before and after SRS. The VAS pain scale was chosen, given that the vast majority of centers participating in the RSSearch® elected to utilize VAS scoring when recording outcomes in the database. VAS scores range on a horizontal scale from “no pain” (scored as 0) to “pain as bad as it could be” (scored as 10) [18].

Inclusion criteria for this retrospective study required that patients have information regarding Dmax, both pre-treatment and post-treatment VAS scores, and initial VAS scores ≥ 3. Following these criteria, a retrospective analysis of patients treated from July 2007 to June 2015 with TN1 or TN2 enrolled in the RSSearch® identified 163 total patients (125 patients with TN1 and 38 patients with TN2) treated at 15 centers in the US and one center in Australia.

The initial response was defined as patients reporting a lower VAS score after SRS as compared to their pre-treatment VAS score. The majority of patients reported only one follow-up less than one-year post-treatment; for these patients, the post-treatment VAS score recorded at this visit was the one used for our analysis. If patients reported more than one follow-up less than one year after SRS, then the lowest post-treatment VAS score reported among all follow-ups was utilized to examine initial pain relief. To assess the effectiveness of SRS in providing initial pain relief, two paired t-tests were run for both TN1 and TN2 cases comparing pre-treatment and post-treatment VAS scores. Logistic regressions (or Fisher's exact test when logistic regression was not feasible) were used to screen for potential prognostic factors for initial pain relief and to examine the relationship between Dmax and toxicity incidence.

Regarding long-term pain relief, a patient-reported VAS score > 1 at one-year follow-up or greater was defined as failure of sustained pain relief. Twenty-eight TN1 patients had a recorded VAS score one year or greater after being treated with SRS (97 TN1 patients and 32 TN2 patients had their last follow-up reported less than one year following treatment and were not included in further analysis). Potential prognostic factors relating to sustained pain relief were evaluated using the Kaplan-Meier method and continuous log-rank analysis. 

All statistical analysis was performed using Stata 14.0 (StataCorp, College Station, TX).

## Results

### Patient demographics and lesion characteristics

Table 1 summarizes both patient demographics and lesion characteristics of our cohort. Females were found to constitute the majority of both TN1 and TN2 populations, representing 74% and 63% of cases, respectively. The average age of TN1 (68.2 years) and TN2 (66.7 years) patients were similar. The vast majority of patients with TN1 were Caucasian (88%), whereas, in TN2 cases, the majority (58%) did not have their race noted. Roughly half of both TN1 and TN2 patients were previously reported as using medications prior to CK SRS, although it is likely that a greater proportion of patients used medications prior to CK SRS as most TN patients turning to SRS are refractory to pharmacotherapy. Median lesion volumes for TN1 and TN2 cases studied were 0.085 cc and 0.107 cc, respectively. Trigeminal nerve lesions were more common on the right-side for TN1 (58.7%) and TN2 (53.8%) patients. 

**Table 1:** Patient Demographics and Lesion Characteristics

Variable	TN1 Cases (n = 125)	TN2 Cases (n = 38)
Male	26%	37%
Female	74%	63%
Mean Age (range)	68.2 years (29-100)	66.7 years (32-96)
Median Initial KPS (range)	90% (50% - 100%)	80% (60% - 100%)
Race		
	Caucasian - 88%	Caucasian - 42%
	African-American - 5%	African-American - 0%
	Hispanic - 0.7%	Hispanic - 0%
	Pacific/Asian-Islander - 0.7%	Pacific/Asian-Islander - 0%
	Unknown - 5.6%	Unknown - 58%
Prior Treatments		
	Medications - 47%	Medications - 53%
	Rhizotomy - 0.6%	Rhizotomy - 1.7%
	Radiofrequency Ablation - 0.6%	Radiofrequency Ablation - 1.7%
	Trigger Point Injection - 0%	Trigger Point Injection - 1.7%
	Nerve Blocks - 0.6%	Nerve Blocks - 0%
	*Not Specified *- 51.2%	*Not Specified *- 58.1%
Median lesion volume (cc) (range)	0.085 (0.018 - 0.461)	0.107 (0.037 - 0.33)
Right trigeminal nerve lesion	58.7%	53.8%
Left trigeminal nerve lesion	41.3%	46.2%

### Initial pain relief

Treatment characteristics and changes in VAS scores were examined. The results of this analysis can be found in Table 2. Median times to the last recorded follow-up for TN1 and TN2 patients were four months (range: 0.25 - 44 months) and five months (range: 0.25 - 41 months), respectively. Median Dmax for both cohorts was 75 Gy with a larger range for TN1 patients (67.42 Gy - 110.29 Gy) as compared to TN2 patients (70 Gy - 78.48 Gy). Mean pre-treatment VAS scores were 7.47 and 6.89 for TN1 and TN2 patients, respectively. Following CK SRS, mean VAS scores declined in both TN1 (1.86) and TN2 (1.84) cases. The differences between pre-treatment and post-treatment VAS scores were significantly different (p < 0.0001) for both TN1 and TN2 cases following paired t-test.

**Table 2:** Treatment Parameters and Outcomes

Variable	TN1 Cases (n = 125)	TN2 Cases (n = 38)
Median Prescription Dose (Gy) (range)	60.00 (49.00 - 75.00)	73.50 (47.78 - 75.00)
Median Dmax (Gy) (range)	75.00 (67.42 - 110.29)	75.00 (70.00 - 78.48)
Median Isodose (range)	80% (55% - 100%)	100% (68.3% - 100%)
Median Time to Follow-up (range)	4 months (0.25 - 44 months)	5 months (0.25 - 41 months)
Mean pre-treatment VAS score (95% CI)	7.47 (7.00 - 7.94)	6.89 (5.93 - 7.86)
Mean post-treatment VAS score (95% CI)	1.86 (1.33 - 2.38)	1.84 (0.82 - 2.87)
Mean VAS score decline (95% CI)	5.62 (4.93 - 6.32)	5.05 (3.82 - 6.29)
Patients with declines in VAS scores	87.2%	86.8%
Patients with no change in VAS scores	5.6%	7.9%
Patients with increases in VAS scores	7.2%	5.3%

**Figure 1:**
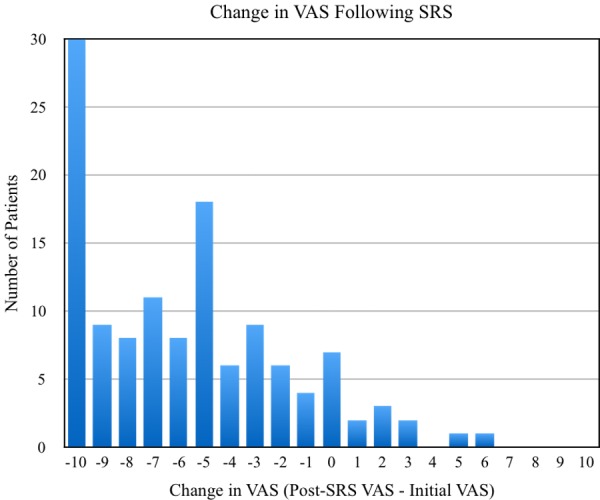
Distribution of changes in VAS scores among TN1 patients following SRS

**Figure 2:**
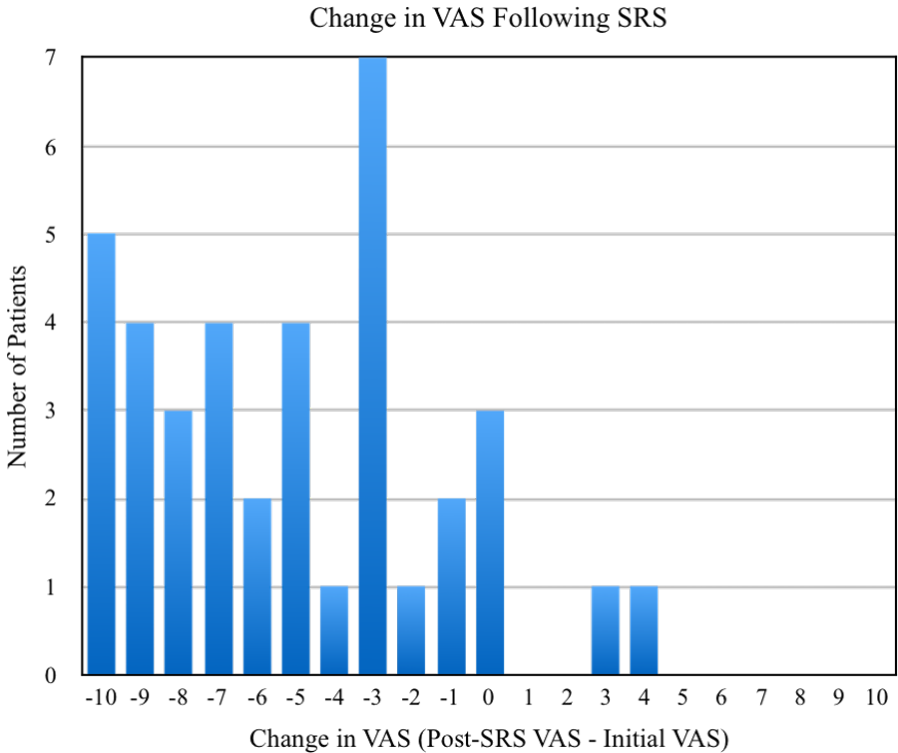
Distribution of changes in VAS scores among TN2 patients following SRS

CK SRS was successful in achieving initial pain relief in 87.2% of TN1 cases and 86.8% of TN2 cases. Roughly 6% and 8% of TN1 and TN2 patients, respectively, experienced no subsequent change in VAS score, and 7.2% and 5.3% of these same populations had pain progression at their last reported follow-up. Average declines in VAS scores were 5.62 (95% CI: 4.93 - 6.32) for TN1 cases and 5.05 (95% CI: 3.82 - 6.29) for TN2 cases, with the distribution of changes in VAS scores (post-treatment VAS - pre-treatment VAS) following CK SRS shown in Figures 1-2. Approximately 42% of patients reported using medications after treatment with CK SRS with the remaining 58% not specifying whether they utilized pharmacotherapy.

We next moved on to identify potential prognostic factors regarding initial treatment response. Factors, such as initial Karnofsky Performance Score (KPS), initial VAS score, age, gender, and Dmax, were examined via univariate logistic regressions. The results of these findings can be found in Tables 3-4 for TN1 and TN2 cases, respectively. For TN1 patients, a higher initial VAS score was associated with a greater likelihood of treatment success (p = 0.015). No treatment or patient characteristics evaluated for TN2 patients were found to significantly impact the likelihood of initial pain relief following CK SRS.

**Table 3:** Potential Prognostic Factors for Initial Response in TN1 Cases

Variable		Success	Failure	p-value
Age				0.103
	Age ≥ 70	65	6	
	Age < 70	44	10	
Gender				0.504
	Female	80	13	
	Male	29	3	
Initial KPS				0.860
	Initial KPS ≥ 80%	97	14	
	Initial KPS < 80%	12	2	
Initial VAS				0.015
	Initial VAS ≥ 7	65	4	
	Initial VAS < 7	44	12	
Dmax				0.372
	Dmax > 75 Gy	47	5	
	Dmax ≤ 75 Gy	62	11	

**Table 4:** Potential Prognostic Factors for Initial Response in TN2 Cases

Variable		Success	Failure	p-value
Age				0.210
	Age ≥ 70	10	3	
	Age < 70	23	2	
Gender				0.488
	Female	21	4	
	Male	12	1	
Initial KPS				0.471
	Initial KPS ≥ 80%	30	4	
	Initial KPS < 80%	3	1	
Initial VAS				0.638
	Initial VAS ≥ 7	16	3	
	Initial VAS < 7	17	2	
Dmax				
	Dmax > 75 Gy	6	2	0.281
	Dmax ≤ 75 Gy	27	3	

### Sustained pain relief

**Figure 3:**
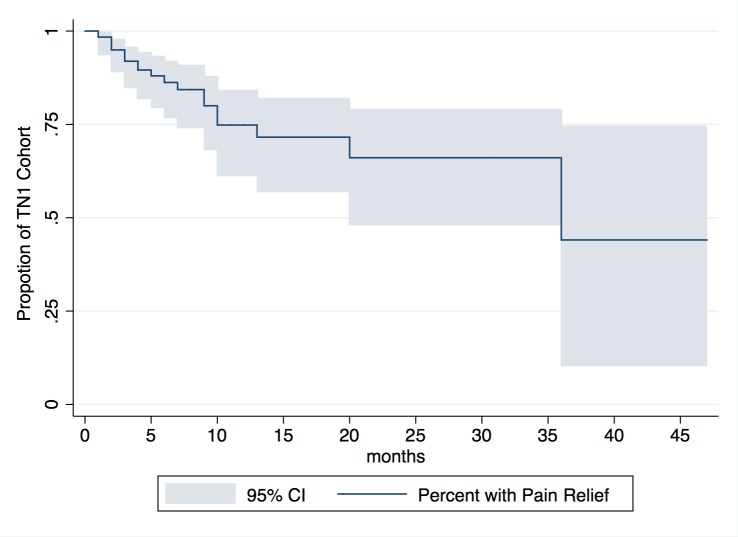
Kaplan-Meier Curve for Sustained Pain Relief Following SRS

We also examined how effective CK SRS was in providing relief of TN-related symptoms for patients reporting follow-ups of one year and greater and if any prognostic factors could predict longer term treatment success. The Kaplan-Meier curve of TN1 patients who reported follow-ups greater than one year can be found in Figure 3. Twenty-eight TN1 patients had follow-ups of greater than one year, and 23 of 28 patients (82%) reported a VAS of 1 or lower one year after treatment. Similarly, 11 patients had follow-ups of greater than two years after CK SRS and eight of 11 patients (72%) reported a VAS of 1 or lower two years after treatment. Only five patients had followed up three years after CK SRS, and two patients (40%) reported lasting pain relief. 

In Table 5, one can find potential prognostic factors relating to sustained pain relief using the Kaplan-Meier method and continuous log-rank analysis. None of the prognostic factors evaluated (age, gender, initial KPS, initial VAS score, and Dmax) were associated with a significantly different likelihood of long-term pain reduction.

**Table 5:** Potential Prognostic Factors for Sustained Pain Relief (Kaplan-Meier Analysis)

Variable	p-Value
Age ≥ 70	0.6042
Gender	0.9748
Initial KPS ≥ 80%	0.7681
Initial VAS ≥ 7	0.2616
Dmax > 75 Gy	0.4548

Six TN2 patients had follow-ups of greater than one year, and 2/6 TN2 patients reported VAS scores lower than pre-treatment assessments. At the two-year follow-up, 0/3 TN2 patients had lasting pain relief. Given the inadequate number of patients that had follow-ups in the TN2 cohort, a Kaplan-Meier analysis was not performed.

### Toxicities

Treatment-related side effects were also examined. A summary of specific toxicities can be found in Table 6. Twenty-three TN1 patients (18.4%) and four TN2 patients (10.5%) had documented side effects following treatment. All acute toxicities reported in the cohort were either RTOG Grade 1 (16/27 patients) or Grade 2 (11/27 patients). The most common side effect reported in both TN populations was sensory neuropathy (facial numbness). Among both TN1 and TN2 cases, other common adverse effects included generalized pain, nausea, hearing loss, and urticaria.

**Table 6:** Summary of Toxicities Note that individual patients may have reported more than one treatment-related side effect.

	TN1 Cases (n = 125)	TN2 Cases (n = 38)
Proportion of patients reporting toxicities	18.4% (23 patients)	10.5% (4 patients)
Number of patients reporting specific toxicities	Neuropathy - 8	Neuropathy - 3
	Pain - 4	Pain - 1
	Nausea- 3	Nausea - 1
	Hearing Loss - 1	Hearing Loss - 0
	Urticaria - 1	Urticaria - 1
	Other - 7	Other - 2

Using univariate logistic regression, we examined whether patients treated with Dmax > 75 Gy had a higher likelihood of experiencing treatment-related toxicities. In TN1 cases, we found that Dmax was not a predictor of toxicity incidence (p = 0.597). However, for TN2 cases, we were unable to run the regression as all four patients that had reported acute toxicities were treated with radiotherapy plans of Dmax > 75 Gy (of eight total patients treated with Dmax > 75 Gy). TN2 patients that were treated with Dmax ≤ 75 Gy reported no acute toxicities (p = 0.0009 following Fisher's exact test).

## Discussion

TN is a chronic disease, and for many patients, pharmacotherapy may not provide therapeutic relief of symptoms. As such, patients have a variety of treatment options, notably rhizotomies, MVD, and SRS. SRS is a non-invasive alternative for TN treatment and may be especially helpful for patients who are unable to undergo anesthesia for MVD or would prefer not to undergo elective surgery. Notably, previous studies have found that CK SRS may be more cost-effective than MVD (given longer hospital stays following MVD) with similar clinical effectiveness with regards to initial pain relief [19].

This retrospective study included 163 TN patients (125 TN1 patients and 38 TN2 patients) with initial VAS scores ≥ 3. Our study is unique in its multi-institutional observation of both TN1 and TN2 patients, the exclusive use of the CyberKnife® Radiosurgery system, and examination of treatment outcomes in community settings. Consistent with the literature, TN was more common in older female patients [1]. Median Dmax for TN1 and TN2 cases were both 75 Gy, which is consistent with median maximal doses previously reported in retrospective studies [20]. Of note is the finding that patients treated with plans with a median Dmax of 78 Gy (range: 70 - 85.4 Gy) had a greater likelihood of initial and longer-lasting pain relief as compared to those receiving SRS with median maximal doses of 75 Gy [20]. However, our study did not identify Dmax > 75 Gy to be correlated with better outcomes for either initial or sustained pain relief in either cohort. 

With regard to initial pain relief, our analysis found that roughly 87% of both TN1 and TN2 patients responded favorably to CK SRS. These results seem to fall in line with the literature. Short-term response rates in studies utilizing CK SRS have historically ranged from 67% to 88% [20-22]. GK SRS studies have also demonstrated the proportion of patients experiencing pain relief fell between 58.5% to 88% [23-24]. Of the prognostic factors examined via logistic regression for initial pain relief, higher initial VAS scores (p = 0.015) were correlated with a higher likelihood of treatment success for TN1 patients. However, no prognostic factors were identified for TN2 patients.

We also examined long-term pain relief following CK SRS for TN1 patients. Twenty-three of 28 patients (82%) reported a VAS score of 1 or less at one-year follow-up, and eight of 11 patients (72%) reported a VAS score of 1 or less at the two-year follow-up. Two of five patients (40%) of the TN1 cohort reported lasting pain relief at the three-year follow-up. However, estimates of long-term pain relief at the three-year follow-up are quite unreliable given the very low sample size (95% CI for pain relief at three years ranged from 10% - 75%) and, thus, should be cautiously interpreted. This is especially relevant given the retrospective nature of our study and the possibility that patients may be more likely to return for subsequent follow-ups due to the lack of sustained pain relief. As such, our findings may underestimate the long-term therapeutic benefit of CK SRS. Our findings at the two-year follow-up are similar to the findings of Karam, et al., who found that at the 28 month follow-up 72% of TN patients treated with CK SRS had freedom from severe pain (defined as Barrow Neurological Institute pain scores of greater than III) following a mean prescription dose of 64 Gy [25]. Of note also is a recent study examining long-term outcomes of GK SRS that found that 77.9%, 73.8%, 68%, and 51.5% of patients remained pain-free without medications at three, five, seven, and 10-year follow-up, respectively [26].  

We identified no treatment or patient characteristics that could predict a greater likelihood of longer-term pain relief following treatment. Of the prognostic factors examined regarding long-term pain relief by Karam, et al., diabetic patients were found to have significantly poorer outcomes (age, gender, or prescription dose were not found to have an impact) [25]. Similarly, Lucas, et al. also noted diabetes to be a poor prognostic factor following GK SRS while other factors examined (i.e., multiple sclerosis, age, duration of symptoms, or radiation dose) were also found to be insignificant [27]. As no data was available on patient comorbidities, we were unable to evaluate the impact of diabetes on the likelihood of treatment success. Given these congruent findings regarding diabetes as a poor prognostic factor and no clear mechanism elucidated thus far, further studies examining this relationship are warranted. 

Roughly 18% and 11% of TN1 and TN2 patients, respectively, reported acute toxicities with sensory neuropathy (particularly facial numbness) being the most common in both cohorts. Similar to other studies, no major adverse effects were noted following CK SRS, with all acute toxicities reported being either RTOG Grade 1 or Grade 2. Other CK and GK SRS studies have noted hypesthesia and facial numbness to be the most common complications in anywhere from 10–50% of patients [20-21, 28-30]. Of note, Dmax > 75 Gy was not a predictor of toxicity incidence in TN1 cases (p = 0.597). However, for TN2 cases, Dmax > 75 Gy was significantly associated with toxicity incidence (p = 0.0009).

There are several limitations to our study that merit mentioning. First, the retrospective nature of this analysis, as well as the low sample size of patients that had long-term follow-up, lowers the power of our findings. This was particularly true for our TN2 cohort, as we were unable to perform a Kaplan-Meier analysis to examine prognostic factors for sustained pain relief, given the lack of follow-up after one year for the vast majority of patients. Another limitation of our study is the potential for underreporting, as may be the case with side-effects, again due to lack of routine long-term follow-up. We are also unable to discuss the mean latency to response as has been done by other studies in the literature, given a lack of uniform and standard follow-up of patients in our cohort. With regards to measurement of pain, we ideally would have used the Barrow Neurological Institute (BNI) pain intensity scoring. However, a very limited number of patients (six TN1 patients and one TN2 patient) reported both pre-treatment and post-treatment BNI scores. Though it is a validated tool for quantification of pain, VAS scoring is inherently a subjective outcome measure as it relies on and is susceptible to individual interpretation and variation. However, given the fact that VAS scores were only compared for each individual patient as opposed to comparing VAS scores between different patients, the subjective nature of the pain scoring is less concerning. Finally, no information was available regarding patient comorbidities (notably, diabetes, as documented in other studies) that could have been examined to better define and characterize prognostic factors.

## Conclusions

This study demonstrates the effectiveness of CK SRS in providing safe and effective pain relief as an alternative to invasive surgical procedures following medication failure with no major adverse toxicities reported. Prospective studies are warranted to determine prognostic factors regarding long-term pain relief following CK SRS as well as better defining optimal radiotherapy planning and dosing.
